# Accuracy of Figure Memory Test of the Brief Cognitive Screening Battery to predict amyloid status in older adults with Subjective Cognitive Decline

**DOI:** 10.1002/alz.092778

**Published:** 2025-01-03

**Authors:** Adalberto Studart Neto, Artur Martins Coutinho, Camila de Godoi Carneiro, Natália Cristina Moraes, Raphael Ribeiro Spera, Monica Sanches Yassuda, Sonia Maria Dozzi Brucki, Carlos Alberto Buchpiguel, Ricardo Nitrini

**Affiliations:** ^1^ University of São Paulo Medical School, São Paulo Brazil; ^2^ University of São Paulo Medical School, São Paulo, São Paulo Brazil; ^3^ Center of Nuclear Medicine, Institute of Radiology, Hospital das Clínicas, Faculdade de Medicina da Universidade de São Paulo, São Paulo, São Paulo Brazil; ^4^ Medical School of University of São Paulo, São Paulo Brazil; ^5^ Medical School of University of São Paulo, São Paulo, São Paulo Brazil; ^6^ University of São Paulo, SAO PAULO, SAO PAULO Brazil; ^7^ Hospital das Clínicas, Faculdade de Medicina da Universidade de São Paulo, São Paulo, São Paulo Brazil; ^8^ Universidade de Sao Pablo, Sao Pablo Brazil

## Abstract

**Background:**

Some older adults with subjective decline (SCD) had a positive amyloid biomarker indicating a preclinical stage of Alzheimer’s disease.

**Objectives:**

To assess the accuracy of Delayed Recall of Figure Memory Test (DR‐FMT) of Brief Cognitive Screening Battery to predict amyloid status in SCD older adults.

**Method:**

The sample consisted of 45 older adults classified as SCD and 25 as cognitively unimpaired (mean age of 76.4 and 73.5, respectively, p = 0.138). They were evaluated with BCSB and a standard neuropsychological battery (which includes MMSE, MoCA, RAVLT, Logical Memory and DR of Rey Complex Figure). Subjects were underwent PIB‐PET to assess their amyloid status and images were classified based on visual and semi‐quantitative analyses with 3D‐SSP methodology.

**Result:**

Twelve SCD older adults (27.3%) were positive amyloid against six in the controls (23.1%). In SCD group, DR‐FMT was the only memory test that correlated with SUV in amyloid PET (r = ‐0.514, p < 0.001). Only DR‐FMTshowed significant area under the curve (AUC) in the ROC curve in SCD older adults (AUC = 0.771, 95% CI 0.621 ‐ 0.921). Among SCD older adults, DR‐FMT < 8.0 had a sensitivity of 83.3%, a specificity of 68.7% and an accuracy of 72.7%.

**Conclusion:**

FMT proved to have a good sensitivity and accuracy to predict amyloid status in SCD older adults.

